# Functionally linked potassium channel activity in cerebral endothelial and smooth muscle cells is compromised in Alzheimer’s disease

**DOI:** 10.1073/pnas.2204581119

**Published:** 2022-06-21

**Authors:** Jade L. Taylor, Harry A. T. Pritchard, Katy R. Walsh, Patrick Strangward, Claire White, David Hill-Eubanks, Mariam Alakrawi, Grant W. Hennig, Stuart M. Allan, Mark T. Nelson, Adam S. Greenstein

**Affiliations:** ^a^Division of Cardiovascular Sciences, Faculty of Biology, Medicine, and Health, University of Manchester & Manchester University Teaching Hospitals NHS Foundation Trust, Manchester M13 9PL, United Kingdom;; ^b^Geoffrey Jefferson Brain Research Centre, The Manchester Academic Health Science Centre, Northern Care Alliance NHS Group, University of Manchester, Manchester M13 9PL, United Kingdom;; ^c^Division of Neuroscience and Experimental Psychology, School of Biological Sciences, Faculty of Biology, Medicine, and Health, Manchester Academic Health Science Centre, University of Manchester, Manchester M13 9PL, United Kingdom;; ^d^Department of Pharmacology, Larner College of Medicine, University of Vermont, Burlington, VT 05405

**Keywords:** Alzheimer’s, potassium channel, calcium signaling, small artery

## Abstract

The brain microcirculation is increasingly viewed as a potential target for disease-modifying drugs in the treatment of Alzheimer’s disease patients, reflecting a growing appreciation of evidence that cerebral blood flow is compromised in such patients. However, the pathogenic mechanisms in brain resistance arteries underlying blood flow defects have not yet been elucidated. Here we probed the roles of principal vasodilatory pathways in cerebral arteries using the APP23 mouse model of Alzheimer’s disease, in which amyloid precursor protein is increased approximately sevenfold, leading to neuritic plaques and cerebrovascular accumulation of amyloid-β similar to those in patients with Alzheimer’s disease. Pial arteries from APP23 mice (18 mo old) exhibited enhanced pressure-induced (myogenic) constriction because of a profound reduction in ryanodine receptor-mediated, local calcium-release events (“Ca^2+^ sparks”) in arterial smooth muscle cells and a consequent decrease in the activity of large-conductance Ca^2+^-activated K^+^ (BK) channels. The ability of the endothelial cell inward rectifier K^+^ (Kir2.1) channel to cause dilation was also compromised. Acute application of amyloid-β 1-40 peptide to cerebral arteries from wild-type mice partially recapitulated the BK dysfunction seen in APP23 mice but had no effect on Kir2.1 function. If mirrored in human Alzheimer’s disease, these tandem defects in K^+^ channel-mediated vasodilation could account for the clinical cerebrovascular presentation seen in patients: reduced blood flow and crippled functional hyperemia. These data direct future research toward approaches that reverse this dual vascular channel dysfunction, with the ultimate aim of restoring healthy cerebral blood flow and improving clinical outcomes.

## Significance

Patients with Alzheimer’s disease show hypoperfusion of the brain and this may contribute to disease progression. To elucidate underlying mechanisms, we studied pial arteries from 18-mo-old mice with Alzheimer’s disease due to overexpression of amyloid precursor protein. We found enhanced pressure-induced constriction of arteries because of reduction in ryanodine receptor-mediated, local calcium-release events (“Ca^2+^ sparks”) in arterial smooth muscle cells and a consequent decrease in the activity of large-conductance Ca^2+^-activated K^+^ (BK) channels. This phenotype was partially recapitulated by application of an amyloid-β peptide to healthy arteries. Our results will direct further research to restore cerebrovascular function, which is damaged in Alzheimer’s disease, leading to potentially new treatment options.

Small vessel diseases of the brain are increasingly recognized as important contributors to the functional and cognitive decline of patients with Alzheimer’s disease (AD) (1, 2). This concept has particular resonance, given the diverse spectrum of molecular targets within the cerebral microcirculation and the paucity of effective neuronally directed treatments. One clear intersection between small vessel disease of the brain and AD is the small artery condition, cerebral amyloid angiopathy, a form of microvascular dysfunction that is far more prevalent than previously thought and one that effectively coexists with AD (3, 4)

AD and cerebral amyloid angiopathy are also linked from a pathophysiological perspective, with both conditions being characterized by deposition and accumulation of amyloid β (Aβ) peptide. Two isoforms predominate, with the Aβ(1-40) isoform localizing to the walls of small arteries, whereas the Aβ(1-42) isoform is found in neuritic plaques (5). Previous studies have established that Aβ(1-40) constricts healthy cerebral arteries and, when applied exogenously to the brain, the peptide reduces cerebral blood flow (6, 7). It has further been established that exposure to Aβ peptide induces pericyte contraction, which causes capillary constriction (8).

Broadly speaking, the microcirculation of the brain fulfills two key functions: maintenance of blood flow in the face of moment-to-moment changes in blood pressure (cerebral autoregulation) and neuronal activity-dependent increases in local blood flow (functional hyperemia).

In the first of these two functions, cerebral arteries (diameter, 100 to 200 µm) respond to increases in blood pressure with a contractile response that serves to maintain constant flow. One major contributor to cerebral autoregulation is contraction of arterial smooth muscle cells in response to an elevation of pressure, a depolarization-dependent response intrinsic to smooth muscle myocytes termed “myogenic constriction” (9). In addition to triggering constriction, intraluminal pressure acts through a parallel negative feedback pathway that opposes vasoconstriction to further fine-tune the diameter of pressure-constricted arteries. This latter mechanism acts by increasing local Ca^2+^ signals, known as Ca^2+^ sparks, that reflect release of intracellular Ca^2+^ through the opening of a small cluster of ryanodine receptors in the sarcoplasmic reticulum membrane (10). Ca^2+^ sparks, in turn, activate juxtaposed large-conductance Ca^2+^-activated K^+^ (BK) channels in the plasma membrane of vascular smooth muscle cells (VSMCs), resulting in K^+^ efflux, membrane potential hyperpolarization, deactivation of voltage-dependent L-type Ca^2+^ channel-mediated Ca^2+^ influx, and diminished Ca^2+^-dependent contraction.

The second major function of the microcirculation is to direct blood flow to localized areas of heightened neuronal activity. This activity-dependent increase in perfusion, termed functional hyperemia, is mediated by an ensemble of mechanisms referred to as neurovascular coupling. An important element underpinning this process is the capillary endothelial cell (EC) inwardly rectifying K^+^ (Kir2.1) channel (11), which acts as a sensor of K^+^ released into the extracellular space from neurons and astrocytes as a byproduct of neural activity. Activation of Kir2.1 channels in capillary ECs by the resulting (modest) increase in extracellular [K^+^] induces a retrograde (against the flow of blood) membrane potential hyperpolarization that propagates via ECs of the microvasculature (capillary to arteriole to artery) to induce upstream dilation. The resultant increase in local blood flow toward the site of signal initiation thus supports ongoing neural activity (11). Interestingly, this ionic communication is differentially sensitive to disruption in different pathological settings. We have found that in CADASIL, the most common genetic small vessel disease of the brain, this Kir2.1-mediated signaling mechanism is abrogated in capillary ECs but is fully intact in arteriolar ECs (12), whereas it is crippled in both arteriolar and capillary ECs in the context of hypertension, a major driver of more common sporadic small vessel disease of the brain (13). AD appears to be associated with damage to endothelial Kir2.1 function throughout the cerebral microcirculation. Thus, electrical signaling between neurons and capillary ECs is disrupted in the 5xFAD mouse model of AD as a result of compromised capillary Kir2.1 function (14) and a similar pattern of Kir2.1 dysfunction in upstream pial arteries has been described in the J20 mouse model of AD (15).

Here, we tested the hypothesis that overexpression of Aβ in AD directly impacts the function of cerebral resistance arteries. For these studies, we used the APP23 mouse model, which exhibits approximately a sevenfold increase in the expression of amyloid precursor protein, leading to a cerebrovascular phenotype that approximates that of patients with AD and cerebral amyloid angiopathy (2, 3, 16–19). Our findings demonstrate that 18-mo-old APP23 mice exhibit an exaggerated constriction of cerebral resistance arteries with dysfunction of two key vasodilatory mechanisms: Ca^2+^ sparks-to-BK coupling, which is responsible for setting basal arterial tone and therefore blood flow to the brain, and endothelial Kir2.1 activity, which in capillaries is crucial to a major neurovascular coupling mechanism underpinning functional hyperemia. We also show that exposure of cerebral arteries from young, healthy wild-type (WT) mice to Aβ(1-40) peptides disrupts the Ca^2+^ spark–BK channel vasoregulatory mechanism in VSMCs, partially recapitulating the resistance artery dysfunction phenotype seen in the APP23 mouse. From a clinical perspective, both blood flow to the brain and neurovascular coupling are disrupted in patients with AD (20–23). Our data therefore offer a mechanistic explanation for how resistance artery dysfunction develops in AD and suggest novel avenues for future therapeutic interventions designed to restore healthy blood flow to the brain.

## Results

### Amyloid Deposits in the Cerebrovasculature of APP23 Mice.

Amyloid was observed throughout the vasculature, including leptomeningeal vessels, in the cortex of 18-mo-old APP23 mice (*SI Appendix*, Fig. S1). Cerebrovascular amyloid staining patterns in APP23 mice were consistent with previous reports (26) and comparable to those observed in neuropathological examinations of cerebral amyloid angiopathy in patients (3, 4). Amyloid deposition was not detected in age-matched, WT littermate control mice (*SI Appendix*, Fig. S1).

### Arteries from APP23 Mice Exhibit Impaired Ca^2+^ Spark–BK Channel-Mediated Vasodilation.

**Fig. 1.**
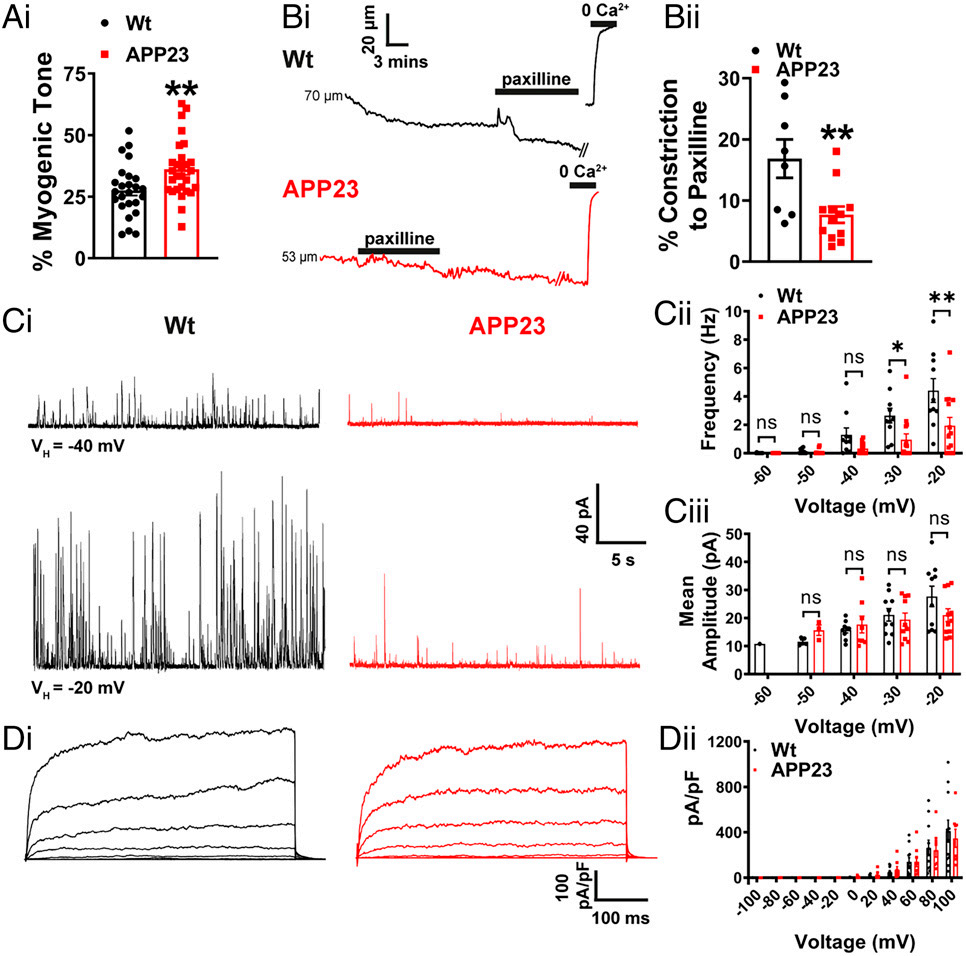
Myogenic tone and BK channel function are impaired in cerebral arteries from APP23 mice. (*A*) Summary data showing that the percent of myogenic tone is significantly higher in cerebral arteries from APP23 mice compared with WT mice (*n* = 25 to 29 arteries, *n* = 15 to 16 mice per group, unpaired *t* test). (*B*, *i*) Representative traces showing the lumen diameter of pressurized cerebral arteries and the contractile response to paxilline (1 μM). (*ii*) Summary data showing a significantly attenuated constriction in response to paxilline in arteries from APP23 mice (red) compared with WT controls (black; *n* = 8 to 12 arteries, *n* = 8 mice per group, *P < 0.01 unpaired *t* test). (*C*, *i*) Representative traces of STOCs recorded using the perforated-patch configuration at −40 mV (*Upper*) and −20 mV (*Lower*) in VSMCs isolated from WT (black) and APP23 (red) cerebral arteries. Summary data over a range of membrane potentials (−60 to −20 mV) showing a lower frequency (*ii*) but no change in mean amplitude (*iii*) of STOCs in VSMCs isolated from APP23 cerebral arteries compared with those from WT controls (*n* = 10 to 14 cells, *n* = 4 to 5 mice per group, *P < 0.05, **P < 0.01, two-way ANOVA with Sidak post hoc test). (*D*, *i*) Representative traces of paxilline-sensitive (1 μM) whole-cell currents in VSMCs isolated from WT (black) and APP23 (red) cerebral arteries obtained using a step protocol from −100 mV to +100 mV in 20-mV steps. (*ii*) Summary data showing no differences in the density of paxilline-sensitive currents between groups at any membrane potential (*n* = 8 to 11 cells, *n* = 4 to 5 mice per group, two-way ANOVA with Sidak post hoc test).

Arteries from APP23 mice exhibited significantly greater constriction in response to intraluminal pressure compared with WT arteries, indicative of enhanced myogenic tone (Fig. 1*A*). To further investigate this enhanced basal pressure-induced constriction in APP23 arteries, we compared the effects of paxilline (1 µM), a selective BK channel blocker, on pressure-constricted arteries from WT and APP23 mice. In WT arteries, bath-applied paxilline induced a stable ∼15% constriction, consistent with previous studies (10). However, the constriction in response to paxilline was significantly blunted in arteries from APP23 mice (Fig. 1*B*), indicating diminished BK channel activity and suggesting that BK channel dysfunction contributes to the increase in the basal tone of APP23 arteries. Next, using a patch-clamp electrophysiology approach, we assessed BK channel function in VSMCs by measuring BK channel-mediated, Ca^2+^ spark-induced outward hyperpolarizing K^+^ currents, which manifest as so-called spontaneous transient outward currents (STOCs) (10). Because BK currents are activated by voltage as well as Ca^2+^, we measured STOCs from VSMCs at voltages between −60 mV and −20 mV using a perforated-patch approach. As shown in Fig. 1*C*, and consistent with our diameter studies, STOC frequency was reduced in VSMCs isolated from pial arteries of APP23 mice compared with those from WT mice. Mean STOC amplitude was unchanged between APP23 and WT VSMC. A reduction in STOC frequency could point to a defect in the BK channel itself or a failure in the Ca^2+^ spark-to-BK channel linkage. To distinguish between these two possibilities, we studied BK channel function directly using the whole-cell configuration of the patch-clamp technique, which bypasses the Ca^2+^ spark-release mechanism and evokes K^+^ currents by voltage at a fixed [Ca^2+^] (i.e., in the pipette solution). BK currents were subsequently isolated using paxilline (1 μM) (Fig. 1 *D*, *i*). Paxilline-sensitive currents did not differ between WT and APP23 pial artery VSMCs (Fig. 1*D*), suggesting that BK channels themselves are unaffected in APP23 mice.

**Fig. 2.**
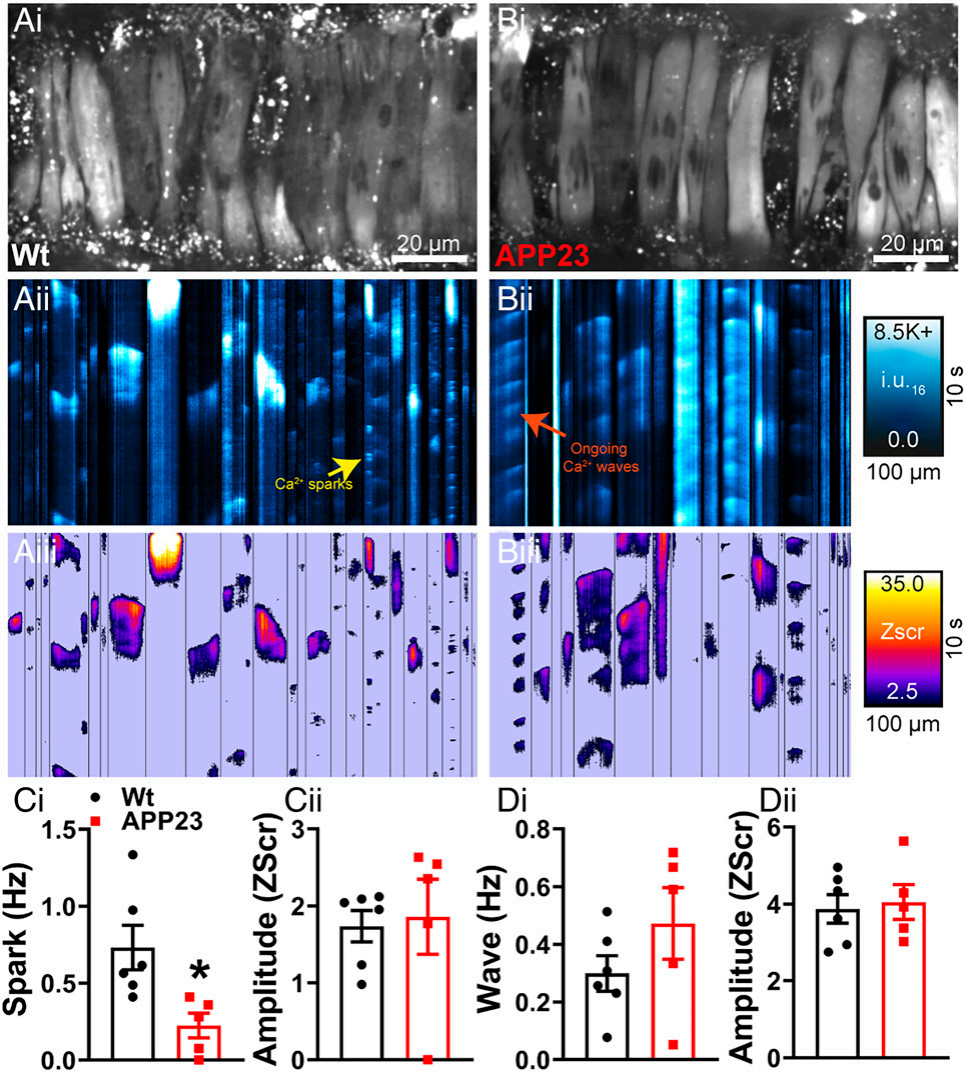
Ca^2+^ spark activity is attenuated in cerebral arteries from APP23 mice. Ca^2+^ signals in pressurized cerebral arteries from WT (*A*) and APP23 mice (*B*). Cerebral arteries were loaded with Fluo-4-AM, pressurized, and imaged using the spinning-disk confocal microscope (*A*, *i* and *B*, *i*). Recordings were processed to generate ST maps (*A*, *ii* and *B*, *ii*), whereby each active site within the imaged region is a column, and the Ca^2+^ events at that site are represented by their spatial spread (width), duration (length), and relative Ca^2+^ release (intensity). Ca^2+^ events were then converted to pixels on ST maps (*A*, *iii* and *B*, *iii*) and given a composite Zscr, allowing individual events to be categorized into Ca^2+^ sparks and waves based on duration (length), spatial spread (width), and intensity. (*C*, *i*) Ca^2+^ spark frequency is significantly reduced in cerebral arteries from APP23 compared to WT, with no differences observed in Ca^2+^ spark event amplitude (Zscr) (*ii*) (*n* = 5 to 6 mice per group, unpaired *t* test). No differences observed in the frequency (*D*, *i*) or amplitude (Zscr) (*ii*) of Ca^2+^ wave events in pressurized cerebral arteries from WT and APP23 mice (*n* = 5 to 6 mice per group, *P < 0.05, unpaired *t* test).

The reduction in STOC frequency in the context of a normal BK channel current-voltage density points to a failure of Ca^2+^ sparks to activate BK channels. We investigated this by imaging Ca^2+^ sparks in intact pressurized arteries loaded with Fluo-4-AM using high-speed spinning-disk confocal microscopy (Fig. 2 *A*, *i* and *B*, *i*). Using our recently developed imaging analysis techniques (27), we converted the recordings into spatiotemporal (ST) maps (Fig. 2 *A*, *ii* and 2 *B*, *ii*) in which each active site within the imaged region is a column, and the Ca^2+^ events at that site are represented by their spatial spread (width), duration (length), and relative Ca^2+^ release (intensity). From here, each Ca^2+^ event was converted into pixels and quantified by calculating an individual *Z*-score (Zscr) based on the average quiescence intensity and SD of the event (*Methods* and Fig. 2 *A*, *iii* and 2 *B*, *iii*). Since each cell has a different background fluorescence depending on the loading of Fluo-4-AM, pixel conversion removes background noise and allows individual events to be categorized into Ca^2+^ sparks or waves based on their duration and spatial spread. Ca^2+^ sparks can be visualized as the repetitive, small dot-like events in ST map columns, whereas Ca^2+^ waves are larger, usually occupying the width of the column (larger spread) and exhibiting a longer duration. Ca^2+^ sparks and interspersed Ca^2+^ waves were clearly detected in cerebral arteries from 18-mo-old WT mice (Fig. 2). However, in APP23 mice, the frequency (reflecting the Zscr) of Ca^2+^ sparks was significantly reduced, with no effect on spark amplitude (Fig. 2*C*, and Movies S1 and S2). There were no alterations in Ca^2+^ wave frequency or amplitude in the APP23 mice compared to WT controls (Fig. 2*D*).

In addition to BK channels, voltage-gated K^+^ (K_v_) channels in VSMCs also regulate the membrane potential, and hence diameter, of resistance arteries. However, in experiments designed to study changes in these channels associated with AD, we found that the K_v_1 channel blocker, 4-aminopyridine (1 mM) produced statistically indistinguishable effects on the diameter of pressure-constricted pial arteries from WT and APP23 mice (*SI Appendix*, Fig. S2), indicating that these channels likely play no role in the increased basal tone in APP23 mice. We did not examine the role of VSMC K_v_2.1 channels (28) to the changes in basal tone in the APP23 mice.

### Endothelial K^+^ Channel-Induced Dilation Is Impaired in APP23 Mice.

**Fig. 3.**
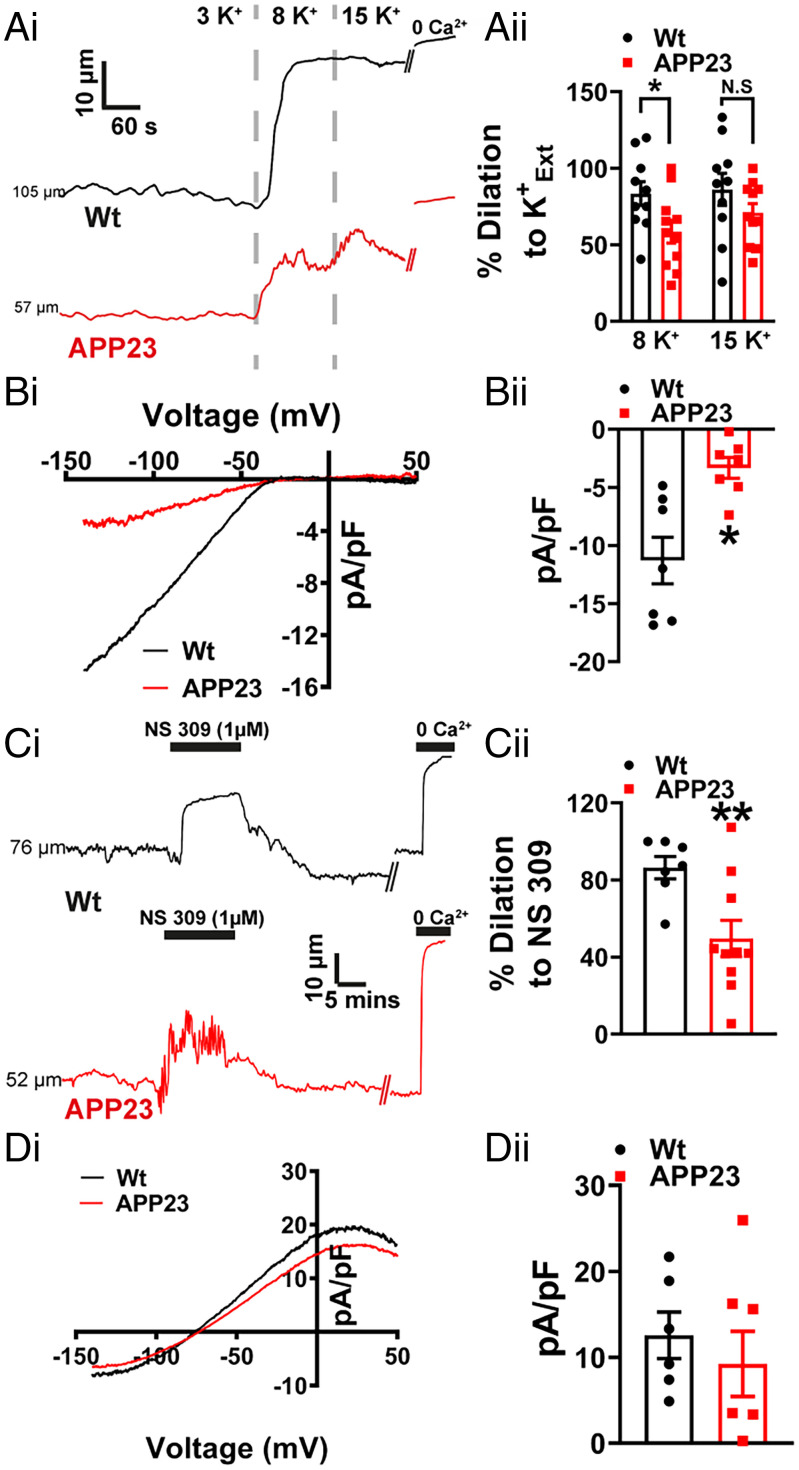
EC Kir2.1 function is impaired in cerebral arteries from APP23 mice. (*A*, *i*) Representative lumen diameter traces from WT (black) and APP23 (red) cerebral arteries, showing dilation to increasing concentration of extracellular K^+^ (K^+^_Ext_). (*ii*) Summary data showing an impaired vasodilation to 8 mM K^+^ but not 15 mM K^+^ in arteries from APP23 mice compared to WT controls (*n* = 10 to 11 arteries, *n* = 6 to 7 mice per group, *P < 0.05, unpaired *t* test). (*B*, *i*) A voltage ramp protocol from −140 mV to +50 mV in high extracellular K^+^ solution (60 mM) and traces shown are the Ba^2+^-sensitive component. (*ii*) Summary data showing a significant impairment of Kir2.1 current density in ECs isolated from APP23 arteries compared to WT controls (*n* = 7 cells, *n* = 4 mice per group, *P < 0.05, unpaired *t* test). (*C*, *i*) Representative lumen diameter traces from WT (black) and APP23 (red) cerebral arteries, showing vasodilation to NS309 (1 μM). (*ii*) Summary data showing impaired vasodilation to NS309 in APP23 cerebral arteries compared to WT controls (*n* = 7 to 10 arteries, *n* = 6 to 7 mice per group, **P < 0.01, unpaired *t* test). (*D*, *i*) Whole-cell IK/SK current induced by the application of NS309 (1 μM) in ECs from APP23 (red) and WT control (black) arteries. Currents were recorded using a ramp protocol from −140 mV to +50 mV. (*ii*) Summary data showing no significant differences in current density of NS309 induced IK/SK currents in EC isolated from APP23 arteries compared to WT controls (*n* = 6 to 7 cells, *n* = 4 mice per group, unpaired *t* test).

We recently showed that K^+^-dependent electrical mechanism responsible for linking neuronal activity with upstream vasodilation is impaired at both capillary and arteriolar levels in a mouse model of hypertension (13) and at the capillary level in the 5xFAD mouse model of AD (14) as a result of compromised Kir2.1 function. Similarly, a recent study of the J20 mouse model of AD found EC Kir2.1 dysfunction in pial arteries from 7- to 10-mo-old mice (15). Here, we investigated Kir2.1 function at the arterial level in WT and APP23 mice by measuring the dilation of isolated, pressure-constricted pial arteries in response to incremental changes in external K^+^. Consistent with the aforementioned study (15), pial arteries from APP23 mice dilated less in response to elevated external K^+^ compared with WT mice (Fig. 3*A*). Using patch-clamp analysis of cerebral artery ECs, we found a significant decrease in Kir2.1 current density in cells from the APP23 mice compared with those from WT mice (Fig. 3*B*), suggesting that this effect is due to Kir2.1 dysfunction.

In addition to expressing Kir2.1 channels, ECs cells in the cerebral circulation also express Ca^2+^-activated intermediate-conductance (IK) and small-conductance (SK) K^+^ channels. Activation of SK and IK channels results in a membrane hyperpolarization that is transmitted to VSMCs through gap junctions, causing vasodilation. Thus, SK and IK channels can be regarded as important adjuncts in endothelial-dependent hyperpolarization. Using the SK/IK activator NS309 to assess SK/IK-dependent dilation of pressurized arteries, we observed a reduced dilatory effect in arteries from APP23 mice compared with that in WT mice (Fig. 3*C*). To determine whether changes in SK/IK activity could account for this reduction in vasodilation, we directly measured SK/IK currents using patch-clamp electrophysiology. Interestingly, we found no difference in IK/SK current density at +30 mV between isolated ECs from APP23 and WT mice (Fig. 3*D*). This implies that the diminished vasodilation to NS309 is not attributable to IK/SK channel dysfunction, but can instead be explained by the loss of endothelial Kir2.1 channels, which would eliminate the amplifying effect of this channel on the hyperpolarizing signal of IK/SK channels (29).

### Acute Exposure of Healthy Arteries to Aβ(1-40) Partially Recapitulates the BK Dysfunction Observed in APP23 Mice.

**Fig. 4.**
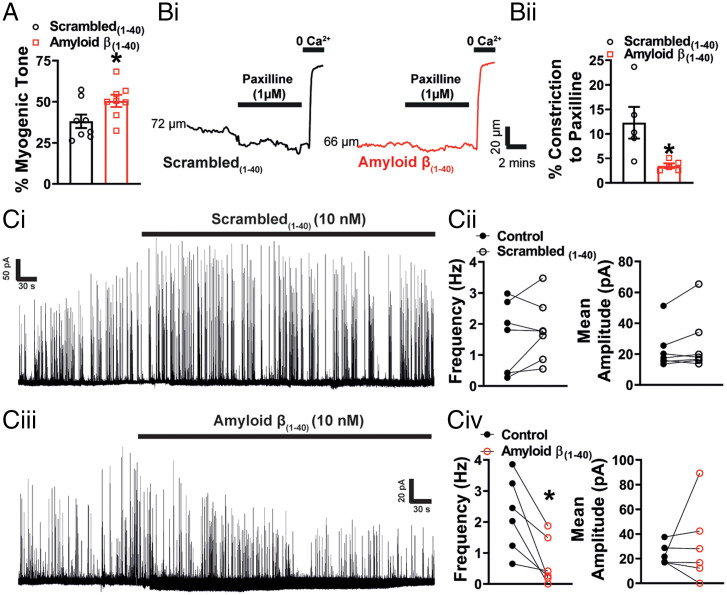
Acute application of Aβ(1-40) peptide impairs BK channel function. (*A*) Summary data showing that pressure-induced constriction of healthy arteries is significantly increased following incubation with Aβ(1-40) (10 nM) compared with those incubated with scambled(1-40) (*n* = 7 to 8 arteries, *n* = 7 to 8 mice per group, *P < 0.05, unpaired *t* test). (*B*, *i*) Representative traces demonstrating degree of constriction to paxilline (1 μM) in pressurized arteries from C57 mice exposed to either scrambled(1-40) or Aβ(1-40) peptides. (*ii*) Response to paxilline was significantly decreased in the presence of Aβ(1-40) compared to scrambled control (*n* = 5 arteries, *n* = 5 mice per group, *P < 0.05, unpaired *t* test). (*C*) Representative traces of STOCs recorded at −30 mV before and after the application of scrambled(1-40) (*i*) or Aβ(1-40) (*iii*) in the presence of BSA (0.1%). The scrambled(1-40) peptide had no effect on STOC frequency or mean amplitude (*ii*) (*n* = 7 cells, *n* = 4 mice, paired *t* test). Aβ(1-40) reduced STOCs frequency had no significant effect on amplitude (*iv*) (*n* = 6 cells, *n* = 4 mice, *P < 0.05, paired *t* test).

**Fig. 5.**
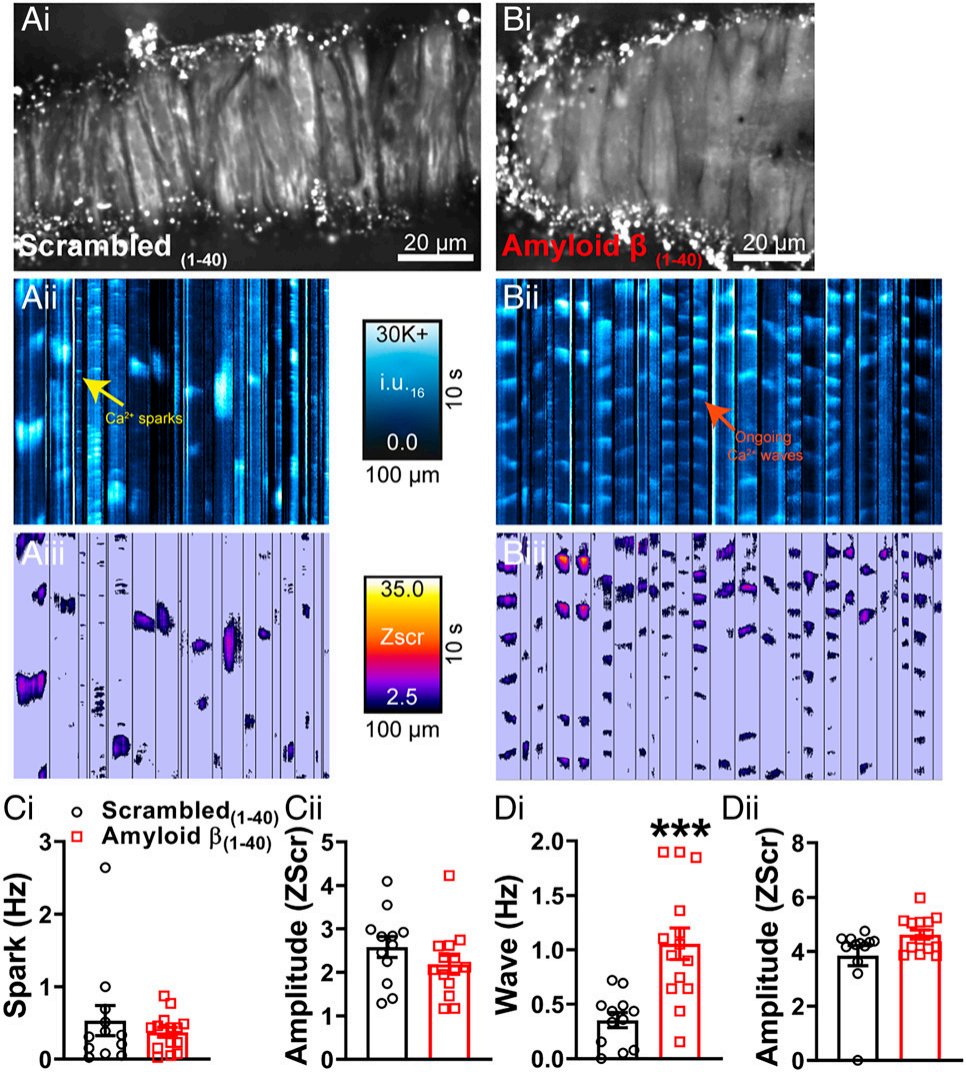
Acute application of Aβ(1-40) peptide generates Ca^2+^ waves. Ca^2+^ signals in pressurized cerebral arteries from C57 mice exposed to scrambled(1-40) (*A*) or Aβ(1-40) (*B*). Cerebral arteries mice were loaded with Fluo-4-AM, pressurized and imaged using the spinning-disk confocal microscope (*A*, *i* and *B*, *i*). Recordings were processed as described in Fig. 2 and *SI Appendix*, *SI Text* before generation of ST maps (*A*, *ii* and *B*, *ii*). Events were then converted to pixels on ST maps (*A*, *iii* and *B*, *iii*) and given a Zscr, allowing individual events to be categorized into Ca^2+^ sparks and Ca^2+^ waves based on duration (length) and spatial spread (width). (*C*, *i*, *ii*) There were no significant differences in Ca^2+^ spark frequency or amplitude (Zscr) between the groups (*n* = 12 to 14 mice per group, ***P < 0.001, unpaired *t* test). Acute application of Aβ(1-40) stimulated a threefold increase in Ca^2+^ wave frequency in pressurized cerebral arteries compared with those observed in the scrambled(1-40) control (*D*, *i*). There was no difference in Ca^2+^ wave amplitude (Zscr) (*ii*) (*n* = 12 to 14 mice per group, unpaired *t* test).

It has been established that Aβ(1-40) has acute effects on cerebral vessels, constricting cerebral resistance arteries (6, 7) and narrowing brain capillaries, the latter of which is caused by contraction of cerebral microvascular pericytes (8). Given the similarity of these reports to our findings in the APP23 mouse, we next studied the mechanisms underlying the vascular effects of Aβ(1-40). Similarly to Niwa et al. (6), we observed a significant increase in healthy mouse pial artery contractile tone following 1-h incubation with Aβ(1-40) (Fig. 4*A*) and, consistent with an effect on BK channel function, a reduction in constriction to paxilline (Fig. 4 *B*, *i*, *ii*). There was no direct effect of Aβ(1-40) on Kir2.1 function, as evidenced by the preservation of vasodilation to external K^+^ and the absence of a change in the endothelial Kir2.1 current after incubation with the peptide (*SI Appendix*, Fig. S4). Focusing on the BK channel, we examined the effect of Aβ(1-40) on STOCs in VSMCs using patch-clamp electrophysiology. Consistent with the observed increase in pressure-induced constriction of the arteries and the reduction in the response of arteries to paxilline, Aβ(1-40) significantly reduced STOC frequency (Fig. 4*C*). There was no effect on STOC amplitude. Finally, we examined the effect of acute Aβ(1-40) exposure on Ca^2+^ signaling in VSMCs of WT pressurized arteries. ST maps (Fig. 5 *A*, *i*, *ii* and *B*, *ii*) and pixel ST maps (Fig. 5 *A*, *iii* and *B*, *iii*), obtained using the same analysis technique as used for APP23 Ca^2+^ imaging, showed a clear increase in wave activity in vessels exposed to Aβ(1-40) compared with those exposed to the scrambled(1-40) peptide (Fig. 5 *D*, *i* and Movie S3). The waves initiated by Aβ(1-40) exhibited a relatively short duration and occurred in a regular and repetitive manner throughout the recording (Movies S3 and S4), an unusual phenotype not previously described in VSMC. There was no difference in the amplitude (Zscr) of waves in Aβ(1-40)–treated arteries compared with scrambled peptide-treated arteries (Fig. 5 *D*, *ii*), and no significant differences in spark frequency or amplitude (Fig. 5*C*) were found in Aβ(1-40)–treated arteries compared with arteries treated with the scrambled peptide.

## Discussion

AD affects one in eight people over 65 y of age, and as the population of the world ages, the lack of effective treatment strategies becomes ever more acute. However, in the last decade—and over 100 y since the disease was first identified—there is growing recognition that a significant degree of the clinical presentation seen in patients with AD reflects progressive cerebrovascular dysfunction due to small vessel disease of the brain (1, 2, 5, 30). Here we report that neuronal overexpression of amyloid precursor protein in the APP23 mouse, an established model of AD, is associated with a distinctive vascular-damage signature in cerebral small arteries. Specifically, we found that a reduction in Ca^2+^ spark frequency in VSMCs and the consequent loss of Ca^2+^ spark-induced BK channel activity is responsible for the enhanced basal constriction of arteries. Additionally, dysfunction of the Kir2.1 channel in the vascular endothelium was associated with compromised vasodilation to external K^+^. Incubation of healthy arteries with the (1-40) isoform of Aβ blunted BK channel function, partially recapitulating the phenotype of arteries from the APP23 mouse.

Overall, our work has several implications. Cerebral hypoperfusion in patients with AD is an established clinical finding, one that is even used to assist in the diagnosis of AD (2, 20–23). Although L-type Ca^2+^ channels and Substance P have been identified in cortical vessels and pericytes associated with amyloid plaques in AD mouse models (31), the mechanisms underlying increases in the contractility of brain arteries have been unclear. One of the principal findings of this study—that loss of Ca^2+^ sparks in VSMCs leads to enhanced pressure-induced constriction of cerebral arteries—could account for the observed cerebral hypoperfusion. To appreciate how alterations in Ca^2+^ spark vasoregulation are linked to changes in brain blood flow, we must consider that, under steady-state conditions, the tone of small resistance arteries exists in a balance determined by two opposing mechanotransduction pathways: constriction in response to pressure (myogenic tone), and the tempering influence of a pressure-sensitive vasodilatory pathway comprising Ca^2+^ spark activation of BK channels (10). Ex vivo preparations of resistance arteries exposed to low intraluminal pressures exhibit Ca^2+^ sparks, but these spark events do not occur at a sufficient frequency to trigger significant BK channel activation. However, increasing intraluminal pressure in resistance arteries to physiological levels (60 mmHg) increases Ca^2+^ spark frequency approximately threefold, an increase sufficient to engage juxtaposed plasma membrane BK channels to oppose membrane depolarization (32, 33). The subsequent hyperpolarization of the cell reduces the activity of voltage-dependent Ca^2+^ channels, thereby decreasing Ca^2+^ levels in the VSMC cytoplasm and lessening the contractile tone of the artery. Alterations in Ca^2+^ spark–BK channel coupling in cerebral arteries, detected as changes in contractility, have been previously described in mouse disease models, notably those for hypertension (34) and muscular dystrophy (35). However, this description of a reduction in Ca^2+^ spark frequency specifically in cerebral arteries from any disease model is unique.

Similarly to the findings from the study examining pial artery function in the J20 mouse model of AD (15), we observed a reduction in pial artery endothelial Kir2.1 function, as evidenced by compromised dilation of resistance arteries in response to external K^+^ together with a decrease in Kir2.1 current density in the ECs of these arteries. Within the capillary network of the brain, the integrity of the EC Kir2.1 channel is critical for efficient neurovascular coupling (11). Increases in extracellular K^+^, which is released by neurons during every action potential, underpin this neurovascular coupling mechanism. Astrocytes, acting as the interface between neurons and capillaries, also release K^+^ upon detection of neuronal activity. Capillary networks detect the resulting increases in extracellular K^+^ through capillary EC Kir2.1 channel activation. The resulting membrane hyperpolarization propagates retrogradely from cell to cell, ultimately reaching the upstream feeding arteriole, where it induces a dilatory response that rapidly increases blood flow to the region of enhanced neuronal activity (11). A failure of this Kir2.1 channel-driven neurovascular coupling mechanism has been documented in a model of CADASIL, the most common genetic form of small vessel disease of the brain (12), as well as in the 5xFAD mouse model of AD (14) and hypertension (13). Taken in context with these studies, our results obtained using the APP23 mouse suggests that AD damages Kir2.1 channel function across the entire cerebrovascular endothelium.

From a mechanistic perspective, we sought to determine whether Aβ could be responsible for the functional damage seen in pial arteries from the APP23 mouse. In the setting of AD, Aβ exists predominantly as two isoforms, Aβ(1-40) and Aβ(1-42), both of which have been implicated in the development of cerebrovascular damage (5). Both Aβ(1-40) and Aβ(1-42) induce endothelin-1 release from brain capillary ECs, and subsequent activation of the cognate receptor initiates contraction of pericytes, causing constriction of cerebral capillaries (8). However, only the Aβ(1-40) isoform is able to constrict myogenically active resistance arteries; this effect is not seen with the Aβ(1-42) isoform (6). Niwa et al. (7) also studied the actions of both Aβ isoforms on cerebral blood flow and showed that cortical superfusion with Aβ(1-40), but not with Aβ(1-42), reduced both functional hyperemia and basal blood flow. The former could be explained by a loss of capillary EC Kir2.1 channels and the latter by a crippled spark–BK pathway.

We hypothesized that the constriction of pial arteries in response to Aβ(1-40) could be due to effects on K^+^ channel vasodilatory pathways, which could account for our observations in the APP23 mouse. Incubation with Aβ(1-40) did indeed affect the BK channel, as evidenced by an increase in pressure-induced constriction, reduction in paxilline-induced constriction, as well as a decrease in the frequency of BK channel-mediated currents (i.e., STOCs). However, in contrast to our findings from APP23 mice, Aβ(1-40) had no effect on Ca^2+^ sparks in VSMCs. Instead, the principal observation was a striking initiation of rhythmic, medium-sized Ca^2+^ waves. Compromised BK channel activity in the presence of normal Ca^2+^ spark activity could be attributable to several mechanisms. BK channels can only be activated by Ca^2+^ sparks in direct proximity to the channel (36). This nanoscale coupling within the VSMC is achieved by the protein junctophilin-2 (36) and STIM1 (37), which tether the sarcoplasmic reticulum to the plasma membrane. Indeed, when the expression of either of these proteins is reduced, Ca^2+^ spark frequency is unchanged but STOCs are reduced and BK vasodilation is impaired (36, 37). Were Aβ(1-40) to interfere with junctophilin-2 or STIM1 function, it could potentially account for BK channel dysfunction in the context of a normal Ca^2+^ spark frequency.

An alternative explanation for the direct effects of Aβ(1-40) on cerebral arteries could be an effect on the BK channel itself. BK channels are regulated both by voltage and Ca^2+^, the latter of which is dependent on functional association of the BK channel β1-subunit (38). If Aβ(1-40) compromised β1-subunit activity, Ca^2+^ sparks would be unable to generate STOCs. A third possibility is that the generation of rhythmic medium-sized waves seen after incubation with Aβ(1-40) represents a failure of sarcoplasmic reticulum function such that, although Ca^2+^ sparks are still generated, their coupling to the BK channel is interrupted. Although generation of rhythmic Ca^2+^ waves in VSMCs is not well described, generation of pathological Ca^2+^ waves within cardiac myocytes is a well-recognized phenomenon that leads to contractile dysfunction and arrhythmias. For example, digitalis increases sarcoplasmic reticulum Ca^2+^ load, leading to Ca^2+^ wave development (39). Conversely, in heart failure, an increase in the “leakiness” of the ryanodine receptor in the sarcoplasmic reticulum leads to pathological Ca^2+^ wave generation and delayed after-depolarization (39, 40).

Within the arteries studied here, how Aβ(1-40) triggered the rhythmic, medium-sized VSMC Ca^2+^ waves and whether this is due to abnormal sarcoplasmic reticulum Ca^2+^ loading or changes in ryanodine receptor open probability, as seen in cardiac pathologies, are intriguing avenues for further investigation. Similarly, it remains unclear how the increase in Ca^2+^ waves induced by exposure of arteries to Aβ(1-40) translates into a reduction in BK channel availability, despite the absence of an effect on Ca^2+^ spark frequency. Furthermore, the phenotype of vascular dysfunction observed as a result of acute administration of Aβ(1-40) differed significantly from that seen in APP23 mice where Ca^2+^ spark frequency was reduced. The reduction in Ca^2+^ spark frequency in cerebral VSMC of the APP23 mice could be accounted for by lower sarcoplasmic reticulum Ca^2+^ stores or defective Ca^2+^ release, potentially implicating the sarcoplasmic reticulum Ca^2+^ ATPase pump or ryanodine receptor function, respectively. Precisely how acute effects of the peptide in triggering pathogenic Ca^2+^ waves in healthy cerebral arteries are converted into the reduction in Ca^2+^ sparks seen in the cerebral arteries from 18-mo-old APP mice remain unclear. Further work is now warranted, potentially involving longer exposure of cerebral arteries to Aβ(1-40), using mice of different ages, or even chronic administration of Aβ(1-40) to induce sarcoplasmic reticulum failure.

Vasodilation in response to direct pharmacological activation of endothelial SK/IK channels was also reduced in APP23 mice compared with WT mice, an observation seemingly at odds with the results of a patch-clamp electrophysiological assessment, which showed that endothelial SK/IK channel current density was equivalent between the two genotypes. This apparent discrepancy between the two approaches for assessing SK/IK channel function can be accounted for by the fact that SK/IK channels and Kir channels are functionally linked, as previously reported (29). Specifically, outward K^+^ currents through SK/IK channels activate endothelial Kir2.1 channels within a functional microdomain, thereby potentiating SK/IK-induced hyperpolarization and boosting vasodilation. Thus, vasodilatory responses to activation of SK/IK reflect the combined action of SK/IK and Kir2.1 channels, whereas electrophysiological recordings of SK/IK currents measure SK/IK activity only. The observed reduction in the dilation of APP23 arteries in response to NS309 can therefore be seen as further evidence of the far-reaching impact that damage to Kir2.1 channel activity has on endothelial—and thus microcirculatory—function.

The APP23 mouse has historically been considered a model of AD with cerebral amyloid angiopathy. Originally described as an uncommon cause of spontaneous intralobar hemorrhage, cerebral amyloid angiopathy is now appreciated as a far more prevalent form of microvascular dysfunction, particularly in the elderly (3). Indeed, in postmortem brain studies, cerebral amyloid angiopathy has been found in up to 90% of patients with AD, indicating that these conditions effectively coexist (3, 4). However, not only is cerebral amyloid angiopathy present in most patients with AD (3), the extent of the angiopathy also correlates with cognitive deficits (41) and the number of white matter hyperintensities (42). It is thus tempting to speculate that targeted and early restoration of microcirculatory homeostasis could improve clinical symptoms in patients with AD. In this regard, recent work suggests that both pathways found to be impaired in small arteries of the APP23 mouse—loss of VSMC Ca^2+^ spark-induced BK channel activity and dysfunction of the Kir2.1 channel in the vascular endothelium—may be amenable to pharmacological rescue. On the endothelial side, we recently reported that retro-orbital application of a water-soluble analog of the membrane phospholipid phosphatidylinositol 4,5-bisphosphate (PIP_2_), which is an essential cofactor for Kir2.1 channels (43), restores Kir2.1 channel-mediated functional hyperemia in mouse models of both CADASIL (12) and AD (14). At the VSMC level, the sensitivity of Ca^2+^ sparks to intraluminal pressure, a defining characteristic of these Ca^2+^ signals, hinges on oxidant activation of cGMP-dependent protein kinase (PKG) (33). Notable in this context is the emergence of novel oxidative activators of PKG (44) that show promise in reversing microvascular pathologies. The frequency of Ca^2+^ sparks in mesenteric VSMCs is also reduced in obesity-related hypertension, where there is a similar increase in pressure-induced constriction of small mesenteric arteries (45). Thus, mechanotransduction of vasodilatory Ca^2+^ signaling in small artery VSMCs may represent a common pathway linking microvascular disease across diverse pathologies.

Overall, our data indicate that overexpression of APP in neurons of the male APP23 mouse leads to dual K^+^ channel deficits in resistance arteries of the brain: an increase in arterial contractility due to a failure of VSMC Ca^2+^ spark–BK channel vasoregulation, and a reduction in external K^+^-induced vasodilation resulting from EC Kir2.1 dysfunction. Although numerous studies have demonstrated small vessel disease of the brain in AD in vivo, ours reports a specific signature of vascular ion channel dysfunction ion within the native resistance arteries in AD. Aβ(1-40) alone also compromises BK channel function, although this appears to have more to do with global dysregulation of Ca^2+^ signaling than an immediate effect on Ca^2+^ sparks. However, pharmacological approaches for restoring Ca^2+^ spark–BK channel vasoregulation, together with restoration of Kir2.1-driven neurovascular coupling using PIP_2_-boosting approaches, may represent a strategy for targeting the two principal cerebrovascular deficits in AD: cerebral hypoperfusion and neurovascular uncoupling. Given that both cerebral hypoperfusion and neurovascular uncoupling are also seen in mild cognitive impairment associated with the prodromal stage of AD (20), early identification of patients at risk could facilitate interventions designed to restore normal function and prove beneficial in preventing the onset or slowing the progression of AD. Finally, our study only examined the cerebral small artery dysfunction in male mice. AD affects more women than men, although women experience a lesser effect on episodic memory (46). In this regard, gender differences in cerebral small artery function will be key to examine in future studies, alongside behavioral approaches.

## Methods

Procedures involving animals were performed in accordance with UK Home Office Guidance on the implementation of the Animals (Scientific Procedures) Act of 1986 under appropriate project license authority and with the approval of the University of Manchester Animal Welfare Ethical Review Board. The APP23 mice used in this study express an isoform of the human *APP* gene containing the Swedish double mutation, APP_751_ *K670N/M671L. Male nullizygous (hereafter WT; *n* = 26) and hemizygous APP23 (hereafter APP23; *n* = 19) mice were allowed to age to 17 to 19 mo prior to experimentation. Homozygous mice die at a young age (3 to 4 mo), and thus cannot be used in an aged protocol. Following killing of the mice, cerebral pial resistance arteries were dissected from the brain and studied using pressure (isobaric) myography and high-speed spinning-disk confocal microscopy. Arteries were digested enzymatically and both VSMCs and ECs were studied using patch-clamp electrophysiology. Brain tissue was also embedded in paraffin prior to immunohistochemistical study of Aβ deposition. Full details of all materials and methods can be found in *SI Appendix*.

## Supplementary Material

Supplementary File

Supplementary File

Supplementary File

Supplementary File

Supplementary File

## Data Availability

All study data are included in the main text and supporting information.
