# Supplementary material for: Functionally linked potassium channel activity in cerebral endothelial and smooth muscle cells is compromised in Alzheimer’s disease
Source: Proc Natl Acad Sci U S A. 2022 Jun 21;119(26):e2204581119. doi: 10.1073/pnas.2204581119 (PMC9245656; doi:10.1073/pnas.2204581119)
Supplement: Supplementary File [file pnas.2204581119.sapp.pdf]

PNAS
www.pnas.org

Supplementary Information for

Functionally linked potassium channel activity in cerebral endothelial and smooth muscle cells is compromised in Alzheimer's Disease

Jade L. Taylor*[1,4], Harry A.T. Pritchard*[1,4], Katy R. Walsh[1,2,4], Patrick Strangward[2,4], Claire White[2,4], David Hill-Eubanks[3], Mariam Alakrawi[1], Grant Hennig[3], Stuart M Allan[2,4], Mark T. Nelson[1,3$], Adam S. Greenstein[1,4].
*Contributed equally

[1]Division of Cardiovascular Sciences, Faculty of Biology, Medicine and Health, University of Manchester & Manchester University Teaching Hospitals NHS Foundation Trust, Manchester, United Kingdom

[2]Division of Neuroscience and Experimental Psychology, School of Biological Sciences, Faculty of Biology, Medicine and Health, Manchester Academic Health Science Centre, The University of Manchester, Oxford Road, Manchester, UK

[3]Department of Pharmacology, Larner College of Medicine, University of Vermont, Burlington, VT, USA

[4]Geoffrey Jefferson Brain Research Centre, The Manchester Academic Health Science Centre, Northern Care Alliance NHS Group, University of Manchester, UK

Corresponding Author:
Professor Mark Nelson (mark.nelson@uvm.edu)

This PDF file includes:

- Supplementary text
- Figures S1 to S4
- Legends for Movies S1 to S4
- SI References

Other supplementary materials for this manuscript include the following:

- Movies S1 to S4

Supplementary Information Text

Methods

Chemicals and reagents were obtained from Merck (Gillingham, UK), unless stated otherwise.

Animal procedures

Procedures involving animals were performed in accordance with U.K. Home Office Guidance on the implementation of the Animals (Scientific Procedures) Act of 1986 under appropriate project license authority and with the approval of the University of Manchester Animal Welfare Ethical Review Board. Mice were euthanized according to the Schedule 1 method (CO_2 overdose followed by exsanguination). The APP23 mice used in this study express an isoform of the human *APP* gene containing the Swedish double mutation, APP_{751} *K670N/M671L. Breeding pairs were sourced from Novartis (Basel, Switzerland) and maintained in-house. Male nullizygous (hereafter Wt; n = 26) and hemizygous APP23 (hereafter APP23; n = 19) mice were allowed to age to 17–19 months prior to experimentation. (Homozygous mice die at a young age (3–4 months), and thus cannot be used in an aged protocol.) Mice were housed under a 12-hour day/night cycle with *ad libitum* access to food and water. After euthanizing mice as described, the brain was isolated into a solution of ice-cold, Ca^{2+}-free, Mg^{2+}-based, physiological saline solution (Mg-PSS) containing 5 mM KCl, 140 mM NaCl, 2 mM $MgCl_2$, 10 mM glucose, and 10 mM HEPES (pH 7.4; NaOH). Cerebral pial resistance arteries were dissected from the brain and stored in this solution on ice. The brain was then cut in the medial plane, and the right hemisphere was fixed in 4% formaldehyde/phosphate-buffered saline (PBS) for 24 hours at 4°C, washed, and stored in 1 x PBS. Commercially available 10-12 week old C57Bl6/j mice (Envigo, UK) were used for the protocols with the amyloid β peptides.

Aβ immunohistochemistry and quantitation

Brain tissue was embedded in paraffin and processed using standard methods. Briefly, 5-µm-thick sagittal tissue sections were dewaxed in xylene and rehydrated through a graded alcohol series to water. The sections were then heated in a water bath at 99°C for 30 minutes in Tris-EDTA, followed by treatment with 97% formic acid for 5 minutes and a thorough wash in Tris buffer. The sections were then incubated overnight at 4°C in a solution of biotinylated Aβ antibody (clone 6e10; Biolegend, UK), diluted 1:100 in Tris-buffered saline containing 0.1% Triton X and 1% bovine serum albumin (BSA). Bound antibody was subsequently visualized by sequential incubation with the streptavidin ABC complex alkaline phosphatase (Vector, UK) and Vector Red substrate system (Vector). Sections were counterstained with hematoxylin (Vector), dehydrated through graded alcohols, cleared in xylene, and coverslip-mounted using DPX mounting agent. Brain sections were imaged with a Nikon ECLIPSE Ci-L microscope using a 10X objective and captured with a DS-Fi3 Microscope (Nikon, UK). Mean CAA score was determined semi-quantitively according to previous methods (1, 2).

Single cell isolation

VSMCs were isolated by digesting cerebral pial arteries in Mg-PSS supplemented with papain (1.0 mg/ml; Worthington Biochemical, NJ, USA), dithioerythritol (1 mg/ml) and BSA (10 mg/ml) at 37°C for 12 minutes, washed three times with Mg-PSS, and then incubated a second time for 14 minutes at 37°C in type II collagenase (1.0 mg/ml; Worthington). Digested arteries were then triturated to liberate VSMCs. Isolated VSMCs were stored in ice-cold Mg-PSS and studied within 6 hours.

Endothelial cells (ECs) were isolated by digesting cerebral pial arteries in EC dispersal physiological saline solution (EC-DPSS) containing 55 mM NaCl, 80 mM Na-glutamate, 6 mM KCl, 2 mM $MgCl_2$, 0.1 mM $CaCl_2$, 4 mM glucose and 10 mM HEPES (pH 7.3; NaOH) supplemented with neutral protease (0.5 mg/ml; Worthington) and elastase (0.5 mg/ml;

Worthington) at 37°C for 57 minutes. Thereafter, collagenase I (0.5 mg/ml; Worthington) was added and vessels were incubated for an additional 3 minutes at 37°C. Vessels were then washed three times with EC-DPSS and triturated to liberate ECs. Isolated ECs were stored in ice-cold EC-DPSS and studied within 6 hours.

Patch-clamp electrophysiology

All currents were recorded using an AxoPatch 200B amplifier equipped with an Axon CV 203BU headstage (Molecular Devices). Currents were filtered at 1 kHz, digitized at 40 kHz, and stored for subsequent analysis. Clampex and Clampfit (version 10.2; Molecular Devices) were used for data acquisition and analysis, respectively. All recordings were performed at room temperature (~22°C). VSMCs or ECs were transferred to a recording chamber and allowed to adhere to glass coverslips for 10–-20 minutes at room temperature. Recording electrodes (3–5 MΩ) were pulled and polished.

Currents were recorded from VSMCs in perforated-patch and ruptured whole-cell patch configurations. For perforated-patch whole-cell recordings, amphotericin B (40 µM) was included in the pipette solution to allow electrical access. Perforation was deemed acceptable if series resistance was less than 40 MΩ. Spontaneous transient outward currents (STOCs) were recorded in a bath solution containing 134 mM NaCl, 6 mM KCl, 1 mM $MgCl_2$, 2 mM $CaCl_2$, 10 mM glucose, and 10 mM Hepes (pH 7.4; NaOH). The pipette solution contained 110 mM K-aspartate, 1 mM $MgCl_2$, 30 mM KCl, 10 mM NaCl, 5 µM EGTA, and 10 mM Hepes (pH 7.2; NaOH). For STOC recordings, VSMCs were voltage-clamped (for 30 seconds) at a range of membrane potentials (-60 to -20 mV). STOCs were analyzed using the threshold method and a frequency was generated at each membrane potential.

BK currents were recorded in the ruptured whole-cell configuration using a step protocol (-100 to +100 mV in 20-mV steps for 500 ms) from a holding potential of −30 mV. Whole-cell BK currents were then isolated from other K^+ currents as a subtraction current before and after the addition of paxilline (1 µM). Paxilline-sensitive current-voltage (I-V) plots were generated using values obtained by averaging the last 50 ms of each voltage step. The VSMC whole-cell bath solution contained 134 mM NaCl, 6 mM KCl, 10 mM glucose, 2 mM $CaCl_2$, 1 mM $MgCl_2$, and 10 mM Hepes (pH 7.4; NaOH). The pipette solution contained 140 mM KCl, 1.9 mM $MgCl_2$, 75 µM Ca^{2+}, 0.1 mM EGTA, and 2 mM Na_2ATP, and 10 mM Hepes (pH 7.2; KOH).

EC currents were recorded in the ruptured whole-cell configuration. All EC currents were recorded in a bath solution containing 134 mM NaCl, 6 mM KCl, 1 mM $MgCl_2$, 2 mM $CaCl_2$, 4 mM glucose, and 10 mM Hepes (pH 7.4; NaOH). The pipette solution contained 10 mM NaOH, 128.6 mM KCl, 11.4 mM KOH, 1.1 mM $MgCl_2$, 2.2 mM $CaCl_2$, 5 mM EGTA, and 10 mM HEPES (pH 7.2; KOH). Kir2.1 currents were initially recorded in the EC bath solution (see above), after which the solution was changed to an equimolar solution containing 60 mM K^+ (NaCl reduced to 80 mM). Kir2.1 currents were recorded using a ramp protocol from -140 to +50 mV over 400 ms, stepping from a holding potential of 0 mV. Kir2.1 currents were then isolated from other currents as a subtraction current before and after the addition of Ba^{2+}. Intermediate (IK)- and small (SK)-conductance Ca^{2+}-activated K^+ currents were recorded in EC bath solution (6 mM KCl external solution). The current induced by application of NS 309 was isolated as the IK/SK current.

Recording and analysis of Ca^{2+} sparks

Cerebral pial arteries were loaded with Ca^{2+}-indicator dye by transferring to Ca^{2+}-free Mg-PSS containing Fluo-4 AM (10 µM) and pluronic acid (0.05%) and incubating in the dark for 30 minutes at room temperature. Arteries were then mounted on glass micropipettes

in an arteriography chamber, pressurized to 60 mmHg, and superfused with imaging physiological saline solution (IPSS; 125 mM NaCl, 3 mM KCl, 26 mM $NaHCO_3$, 1.25 mM $NaH_2PO_4 \cdot H_2O$, 1 mM $MgCl_2$, 4 mM glucose, 2 mM $CaCl_2$), aerated with 5% CO_2/21% O_2 (balance N_2), and warmed to 37°C. Following a 30-minute equilibration period, Fluo-4–loaded arteries were excited by illuminating at 488 nm using a solid-state laser, and fluorescence emission was collected above 510 nm. Images (512 × 512 pixels; 131 × 131 µm) were recorded every 18.9 ms (53 fps) using a 60× water-immersion objective (final magnification, 600×; NA1.2) attached to a Nikon Eclipse TE-2000U microscope.

Ca^{2+} event analysis

Pre-processing

Movies of Ca^{2+} events in isolated arterioles were imported into custom-written software (Volumetry G9e; G.W.H), then debleached (background intensity normalization) and motion-stabilized. The high magnification and acquisition frame rate allow temporal (±1 frame average) and spatial (Gaussian Blur, 3 × 3 pixels; s.d. = 1.0) filtering, reducing noise without loss of Ca^{2+} event details. The variability in the basal fluorescence intensity (F_0) of VSMCs poses problems for the use of traditional F/F_0 amplitude measurements (see Longden et al., 2021). Accordingly, we utilized a new technique that relies on resolving dynamic Ca^{2+} events based on standard deviation at quiescence (SD_q) and Z-score (Zscr)(3). Two variations of this approach were used to isolate dynamic Ca^{2+} events: i) masking active zones and creating spatiotemporal (ST) maps (3), and ii) assembling 3D spatiotemporal Ca^{2+} objects from defined active areas. The second method was used for all quantitative analyses of Ca^{2+} events, but ST maps were created to visually portray changes in Ca^{2+} behavior between mice.

SD_q and Zscr (3D spatiotemporal objects)

Noise was reduced by first decreasing the size of pre-processed movies by half (2 x 2 pixel average), and then the average and standard deviation of the dimmest values experienced at each pixel during the recording (lowest 15% of intensity distribution) were extracted using a quiescence estimator. Each pixel was converted to a Zscr based on the average quiescence intensity and standard deviation, after which a threshold was applied (+2.5 Zscrs) to isolate dynamic Ca^{2+} events. A particle filter was then applied to remove any remaining noise (≤5 pixels in area), and areas with elevated Ca^{2+} were converted to coordinate-based particles (PTCLs) for further refinement.

PTCL analysis

Because small random noise aggregations have a high perimeter:area ratio and their occurrence is mostly limited to a single frame, they can be effectively filtered. After checking the minimum area of the smallest Ca^{2+} events, we applied a second round of size filtering (≤15 pixels). Finally, any remaining particles that did not spatially overlap with particles in the next frame (i.e., noise aggregates) were removed, leaving behind a movie of statistically defined dynamic Ca^{2+} events. The spatial overlap routine (see above) was used to assemble overlapping areas on multiple frames into a 3D spatiotemporal object. All quantitative metrics were extracted from 3D spatiotemporal objects, including duration, size, rates of expansion and contraction, velocity and direction of spread, maximum intensity (Zscr), and a compound variable that is the product of the area, duration and intensity of each Ca^{2+} event, abbreviated AZum^{2}s (absolute Z-score • μm^2 • s). This variable is a good indicator of the total Ca^{2+} output produced by each dynamic Ca^{2+} event into the cytoplasm of a VSMC.

Ca^{2+} spark and wave quantification

All Ca^{2+} signals were collected using the above method and filtered into two main events: Ca^{2+} sparks and Ca^{2+} waves. We defined Ca^{2+} signals with a duration < 0.4 seconds and spatial spread < 5 µm as Ca^{2+} sparks, and those with a duration of 0.5–2 seconds and a spatial spread > 50% of the cell as Ca^{2+} waves.

Pressurized artery diameter measurements and analysis.

Cerebral pial arteries were dissected from the brain and stored in chilled, Ca^{2+} free Mg-PSS. Arterial segments of approximately 50 – 150 µm were mounted on glass pipettes of a similar size in an arteriograph chamber (Living Systems Instrumentation). Arteries were left at 5 mmHg to equilibrate for 15 minutes in pre-warmed (37 °C) physiological saline solution (PSS) of composition; 125 mM NaCl, 3 mM KCl, 26 mM $NaHCO_3$, 1.25 mM $NaH_2PO_4 \cdot H_2O$, 1 mM $MgCl_2$, 4 mM glucose and 2 mM $CaCl_2$, and bubbled with 5% CO_2 in biological air. Luminal diameter was continuously measured throughout the experiment using a camera and edge-detection software (IonOptix, Massachusetts, USA).

After the equilibration period arteries were subjected to an increase in pressure from 5 mmHg to 60 mmHg and left to generate stable myogenic tone. Any arteries that did not generate at least 10% myogenic tone were discarded and a new artery segment mounted. Once stable tone was achieved drugs for determining ion channel activity were applied to the superfusate. Percentage contraction was calculated as degree of contraction relative to passive diameter using the equation: (baseline diameter – contracted diameter) / passive diameter x 100. Percentage dilation was calculated as the full potential dilation for each vessel, calculated using the passive diameter: (active diameter – baseline) / (passive diameter – baseline) x 100. Degree of myogenic tone was calculated as (passive lumen diameter – active lumen diameter / passive lumen diameter) x 100. Passive diameter is determined by relaxing the vessels in a Ca^{2+}-free PSS solution of composition: 119 mM

NaCl, 4.7 mM KCl, 21 mM $NaHCO_3$, 1.18 mM KH_2PO_4, 1.17 mM $MgSO_4$, 3 mM EGTA, 4 mM glucose and 0.01 mM diltiazem.

Statistical analysis

Data are expressed as means ± S.E.M, with values of ‘n’ refer to the number of cells or vessels studied, with ‘N’ the number of animals in each group. Data was deemed normally distributed and compared as indicated in the figure captions using paired or unpaired *t*-tests, or two-way analysis of variance (ANOVA) with a Sidak post hoc test. Cerebrovascular amyloid staining patterns were analyzed using a Mann Whitney test due to lack of detection in the Wt group. Statistical testing was performed using GraphPad Prism software (Version 8.4.3; GraphPad software, San Diego, CA). *P* value of ≤ 0.05 was considered significant, with stars to denote significant differences.

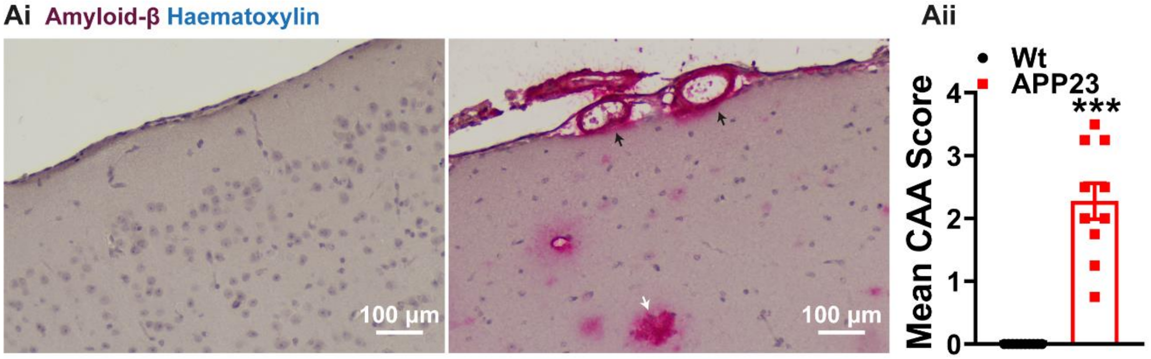

Figure S1. AAP23 mice display a cerebral SVD phenotype.

Brains were dissected out of APP23 mice and wildtype (Wt) litter-mate controls at 18 months of age and amyloid deposition assessed by Aβ immunohistochemistry. (Ai) Representative images of cortex showing the relative presence of cerebrovascular amyloid (black arrows) and amyloid plaques (white arrow) in mice. (Aii) Mean CAA score within the cortex of APP23 and wild type mice (N = 10 mice/group, Mann-Whitney test).

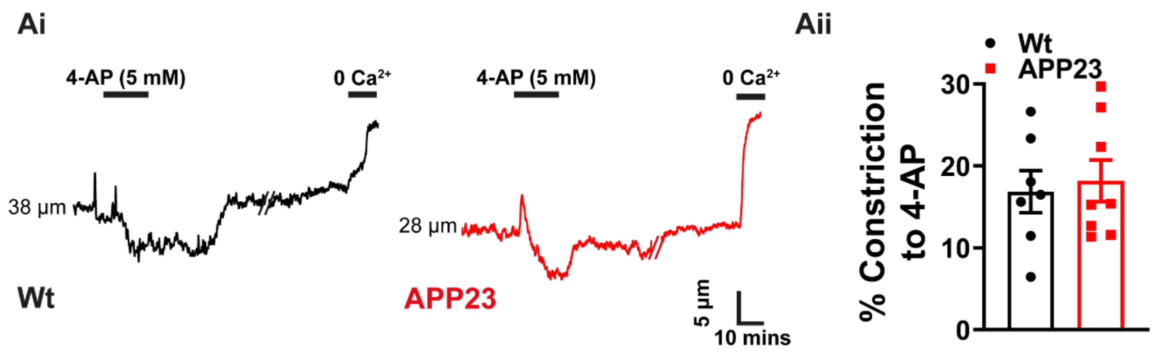

Figure S2. No change in K_v1 channel function in APP23 mice.

Ai) Representative traces of the contraction due to 4-Aminopyridine (4-AP) (5mM) in pressured cerebral arteries from Wt (black trace) and APP23 (red trace). Aii) Summary data showing no difference in the contraction to 4-AP (n = 7-8 arteries, N = 5-6 mice/group, unpaired *t*-test).

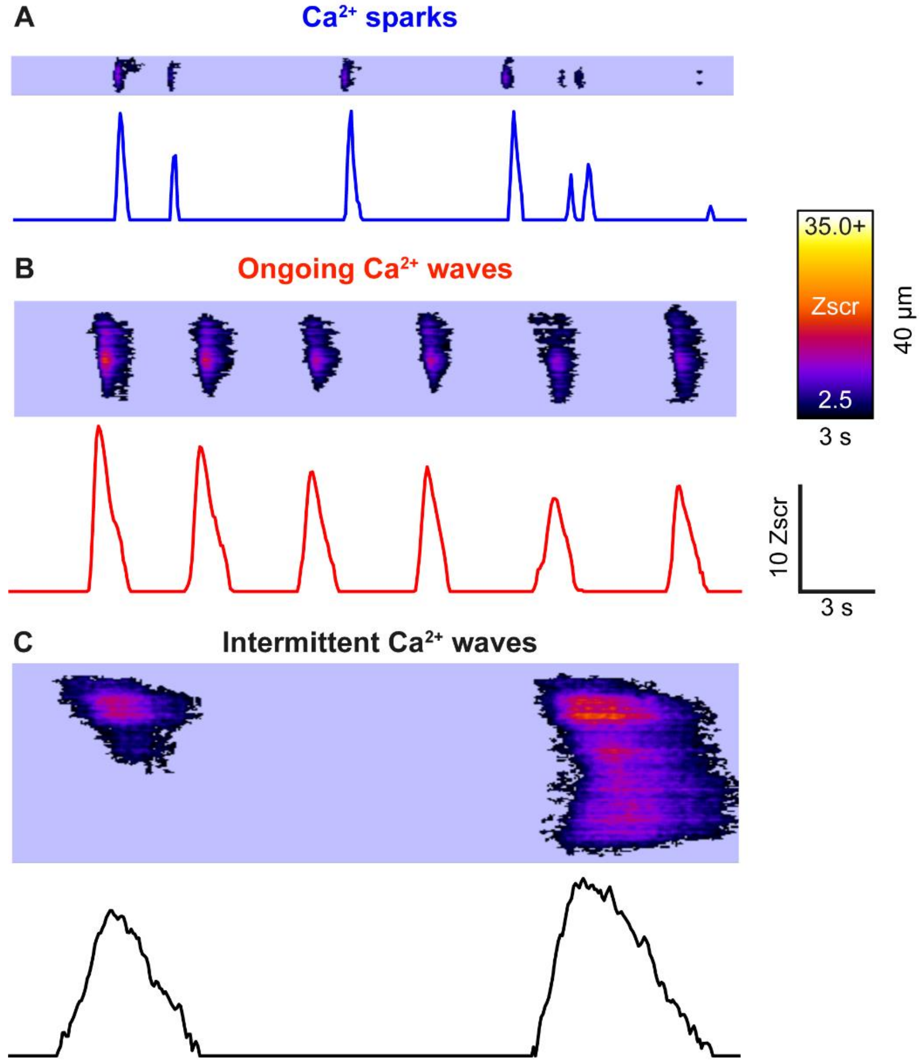

Figure S3. Example traces of different Ca^{2+} signals.

Spatial temporal map with an intensity trace below of Ca^{2+} sparks (A), ongoing Ca^{2+} waves (B) and intermittent Ca^{2+} waves (C), showing the amplitude and rhythmicity of each event.

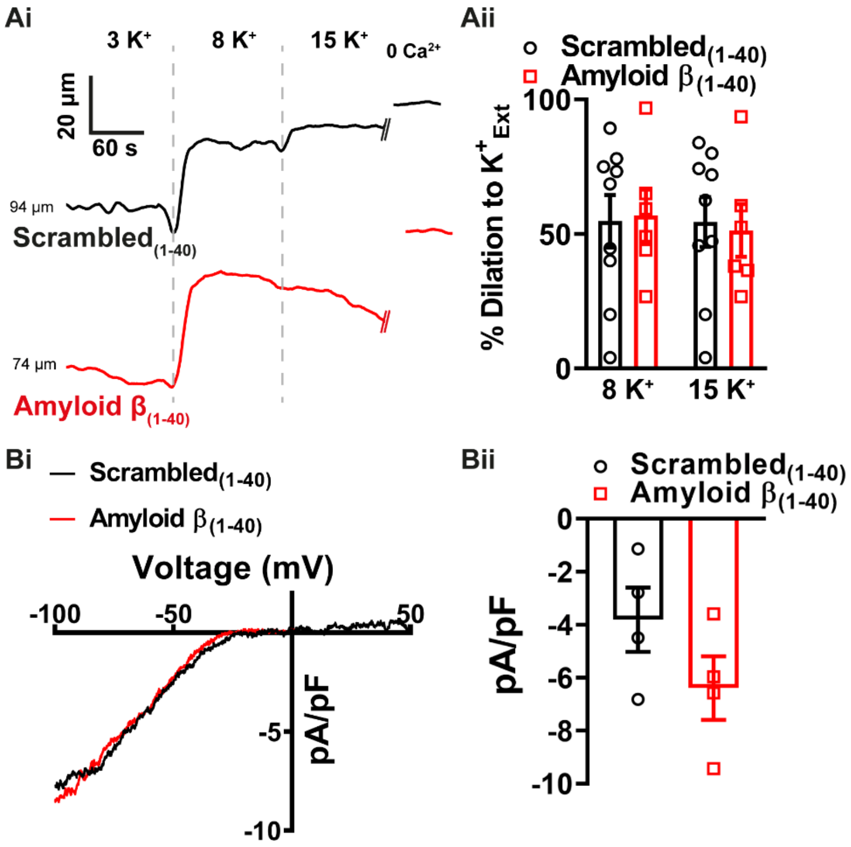

Figure S4. Aβ(1-40) has no acute effect on Kir2.1 channel activity.

Ai) Traces showing arterial diameter changes to increases in extracellular K^+ concentration in scrambled(1-40) (black) and Aβ(1-40) (red). Aii) No differences in the dilation to 8 or 15 mM K^+ (N = 9 arteries, 9 mice/group, unpaired *t*-test). Bi) Cells were exposed to scrambled(1-40) or Aβ(1-40) for 10+ minutes before the Ba^{2+} (100 µM) sensitive currents were recorded from -100 to +50 mV. Bii) There was no significant differences in current density between the groups (n = 4 cells, N = 4 mice/group, unpaired *t*-test).

Movie S1 (separate file)

Representative video showing Ca^{2+} signals in pressurized cerebral artery from 18 month old Wt mouse. Cerebral arteries were loaded with Fluo-4-AM, pressurized and imaged using the spinning-disc confocal microscope (Upper panels). Recordings were processed to generate spatio-temporal (ST) maps (Lower panels), whereby each active site within the imaged region is a column, and the Ca^{2+} events at that site are represented by their spatial spread (width), duration (length) and relative Ca^{2+} release (intensity)

Movie S2 (separate file)

Representative video showing Ca^{2+} signals in pressurized cerebral artery from 18 month old APP23 mouse.

Movie S3 (separate file)

Representative video file of a pial artery from a 12 week old C57Bl6/j mouse.

Movie S4 (separate file)

Representative video file of a pial artery from a 12 week old C57Bl6/j mouse following incubation with amyloidβ (1-40).

.

References

1. J. M. Olichney *et al.*, The apolipoprotein E epsilon 4 allele is associated with increased neuritic plaques and cerebral amyloid angiopathy in Alzheimer's disease and Lewy body variant. *Neurology* **47**, 190-196 (1996).
2. T. Town *et al.*, Blocking TGF-beta-Smad2/3 innate immune signaling mitigates Alzheimer-like pathology. *Nat Med* **14**, 681-687 (2008).
3. T. A. Longden *et al.*, Local IP3 receptor-mediated Ca(2+) signals compound to direct blood flow in brain capillaries. *Sci Adv* **7** (2021).
